# Grounding Cognition in Perceptual Experience

**DOI:** 10.3390/jintelligence12070066

**Published:** 2024-07-10

**Authors:** Ivana Bianchi, Rossana Actis-Grosso, Linden J. Ball

**Affiliations:** 1 Department of Humanities, University of Macerata, 62100 Macerata, Italy; 2 Department of Psychology, University of Milano-Bicocca, 20126 Milano, Italy; rossana.actis@unimib.it; 3 School of Psychology & Humanities, University of Central Lancashire, Fylde Road, Preston PR1 8TY, UK; lball@uclan.ac.uk

The aim of this Special Issue was to put forward a multifaceted reflection on the relevance of perceptual experience in affecting and modeling various aspects of cognitive performance. We sought contributions demonstrating how properties emerging from sensory experience and perceptual organization are of key importance to our mental representations, beliefs, language, imagination, evaluations, actions, and interactions. In our title, we explicitly chose to refer to perceptual experience (i.e., “Grounding cognition in perceptual *experience*”) rather than simply using the more familiar expression “Grounding cognition in perception”. This is because the latter approach has been characterized by a predominant focus on brain activity, whereas our aim here was to complement this valuable mainstream line of research with a different perspective that we also consider valuable. This perspective revolves around the question of what a *phenomenological* approach to investigating the relationship between perception and cognition might be able to contribute to current research. We looked for answers to this question in a broad framework, covering a wide range of topics. How does perceptual experience contribute to the way in which we conceptualize experience? How is perceptual experience reflected in linguistic configurations? In what ways does perception influence people’s judgments, their unfolding reasoning process, and their memories? Each of the 13 papers in this Special Issue provides answers to these questions from a unique point of view.

The importance of phenomenological measurements in investigations of aesthetic appreciation is highlighted in Husselman et al. (2024). This article reveals the value of using subjective metacognitive ratings (e.g., “Did you *feel* you were ‘in the zone’ while you were gazing at the image?”, “How focused did you *feel* while looking at the image?”, “How activated did you *feel* while looking at the image?”, “*How pleasant/enjoyable was it to look* at the image?”…) to complement physiological (EEG) measurement in the study of a complex phenomenon such as aesthetic preference. The article reflects the need to ensure that phenomenological measures remain central in the field of empirical aesthetics so as to capture the processing experience that is integral to aesthetic appreciation. The importance of inserting phenomenological measures into investigations of aesthetic liking resonates with a similar awareness that has emerged in the last decade in research on the “Aha! experience” in problem solving, where self-ratings of pleasure, confidence, suddenness, relief, surprise, drive, and impasse are considered to be critical to understanding the nature of insight (e.g., Ammalainen and Moroshkina 2022; Danek 2023; Danek et al. 2014, 2020). Indeed, this increasing focus on phenomenology reflects a trend that has been emerging across the entire field of thinking and reasoning research in recent years, sparked by Ackerman and Thompson (2017)’s influential position paper on the topic of “meta-reasoning” (see Ackerman 2023; Richardson and Ball 2024; Richardson et al. 2024, for some examples of more recent conceptual developments).

Chiorri and Vannucci (2024) show the pervasiveness of references to phenomenological experience in research concerning autobiographical remembering that has developed over the last three decades, which remains central to current studies and theorizing (Simons et al. 2022; Vanaken et al. 2022; Vannucci et al. 2020, 2021). These authors highlight that the focus of these studies is not on *what* is remembered (i.e., the content of the memory or the number of memories recalled) but on *how* a memory appears in one’s own conscious experience. On the one hand, “how a memory appears” concerns its perceived characteristics, and the authors provide an enlightening picture of the range of dimensions and psychometrically sound instruments that have been developed to explore these characteristics (e.g., vividness, sensory detail, realism, coherence, accessibility, time perspective, visual perspective, emotional intensity, and emotional persistence). On the other hand, however, “how a memory appears” relates to the retrieval processes through which a memory occurs, including how the process of retrieving is experienced by the retriever. In this latter case, the interest concerns the metacognitive aspects of the process of remembering and the epistemic feelings that accompany it, such as fluency and the effort or ease of recall (Moulin et al. 2023). In emphasizing that in recent years, increasing attention has been paid to this second line of research, the authors also point out that a systematic investigation of the phenomenology of different retrieval process is, nevertheless, still missing, as is a suitable methodological toolkit to capture such phenomenology. Importantly, the authors offer promising suggestions to help move investigations forward.

These initial two articles in this Special Issue invite us to reflect on the importance of phenomenology for current analyses of people’s experience of their “internal” world and its associated processing (e.g., relating to memory retrieval, metacognition, and meta-reasoning), whereas several of the subsequent articles focus on the “external” world. Each article adopts a different perspective to highlight the importance of investigating the impact of the phenomenological experience of objects, environments, movements, and images on various types of cognitive performance. As a case in point, Bianchi and Burro (2023) show that a more careful analysis of how the experience of “being different” is phenomenally modulated for perceivers can lead us beyond the “same–different” paradigm as it has been traditionally modeled in psychology, that is dualistically. Perceivers, in fact, spontaneously distinguish three types of non-sameness, that is, similarity, diversity, and opposition, each characterized by a precise perceptual pattern. Acknowledging this phenomenological distinction has many implications for psychological research, as sameness, similarity, diversity, and opposition are basic relationships not only for people’s perception of the external and internal world but also because they are the premise for categorization and therefore the bedrock of language and conceptualization (Goodwin and Johnson-Laird 2005; Halford et al. 2010).

Bertamini (2023) delves into the importance of the experience of *quantity* in modeling people’s understanding of the concept of number, which is a critical area of investigation given the many situations in which we interact with collections of items. The question of whether quantity and numerosity are fundamental properties of our perceptual experience, rather than mere cognitive constructs that emerge in the context of symbolic processing, is not new. The author reviews the current literature concerning three processes (distinct from counting) that have been identified as being related to the perception of numerosity, that is, subitization, estimation and texturization. He also highlights some of the features that underpin the perception of numerosity, which remain unclear and debated within this literature. However, the most original and insightful contributions that can be derived from this article emerge when the author enriches the picture by presenting some observations taken from an old study in the literature on the phenomenal experience of numerosity published in German or Italian and only recently available in translation: Bertamini and Wade (2023) and Bertamini and Bobbio (2024). New and old studies in the literature reveal a substantial difference in perspective. In the current, mainstream approach, accidental features of stimuli (such as the shape of the elements) or of configuration (such as the density and overall area) are dealt with as interfering factors. The unbiased perception of numerosity is seen as normative, and the biases introduced by these accidental features are considered products of perceptual mechanisms not specifically related to the perception of numerosity. Conversely, in the alternative view, which is that suggested by the older, phenomenological literature, these effects are not “problems”. They are not a form of interference, as numerosity in the phenomenal sense is an experience that is intrinsically tied to the Gestalt. This is a change of theoretical perspective that is not only epistemologically interesting but is also one that also has straightforward consequences for the design of experiments.

The benefits of integrating phenomenology into current, mainstream theories are also discussed by Biassoni et al. (2023) in relation to environmental restorativeness theories—that is, the Stress Recovery Theory (Ulrich 1983; Ulrich et al. 1991) and the Attention Restoration Theory (Kaplan 1995; Kaplan and Kaplan 1989). The core aim of this article is to integrate phenomenology and the embodied cognition framework with these two theories. Moving from the general premise that restorativeness arises from a direct encounter between the environment’s phenomenal structure and an individual’s embodied perceptual processes, the authors suggest some points of convergence between the idea of restorativeness as typically operationalized and the idea of tertiary qualities as developed within “experimental phenomenology” (see Bozzi 1990b). The latter offers new lenses to conceptualize the idea of restorativeness as defined within the psycho-evolutionary framework proposed by Ulrich (1983) and Ulrich et al. (1991), that is, as an immediate, unconsciously triggered emotional response that in turn affects arousal levels, attentional processing, and behaviors. Similarly, the factors identified by Kaplan (1995) and Kaplan and Kaplan (1989) as the bases of environmental preference (coherence, legibility, complexity, and mystery), which trigger immediate affective responses and suggest possible ways of interaction compatible with an individual’s needs, can all be conceptualized as phenomenal characteristics and tertiary qualities, as they immediately suggest whether or not an environment appears controllable, supportive, and restorative. Beyond these and other specific aspects discussed in the paper, the overall take-home message is that in the domain of environmental psychology, as in other domains, fresh air seems to come from “including the first-person experience as an essential part of understanding the cognizing being” (Mungan 2023, p.13).

The interaction between the individual and the environment is also at the center of Agostini et al. (2024)’s contribution. In this case, the focus is specifically on motor interaction and on techniques to improve people’s motor performance in sports and motor rehabilitation using acoustic feedback. The authors compare two different techniques to transform movement data into audio signals. In one approach, based on the sonification of movement, physiological and physical data relating to movement are mapped onto psychoacoustic parameters (e.g., loudness, pitch, rhythm). The aim is to offer athletes access to biomechanical information that is otherwise not available to them (Schaffert et al. 2019). In the second auditory modeling approach, the auditory recording of the sounds that are produced by an athlete or a patient during the execution of a movement is used as the model (Agostini et al. 2004). The authors discuss the pros and cons of the two approaches. In relation to the overall aims of this Special Issue, this article represents an example of the profitable application of phenomenology in two, relevant application contexts. Moreover, it makes it clear what it means to be theoretically and methodologically centered on performers’ experience. Indeed, the procedure starts with recording the ecological sound produced during a performer’s execution (and repeated execution) of a gesture that they can hear. The soundtrack associated with the best performance is then selected and used as a model, with the “best performance” being identified through performers’ subjective evaluations of optimal gesture execution, in addition to objective performance outcomes. The soundtrack selected as optimal is then used for training, asking the performer to mentally represent the execution of the movement while listening to the auditory model and, in some cases, where possible, by administering the sound during movement execution. Everything is played within phenomenal boundaries.

Soranzo and Taddio (2023) explore the relationship between neurophysiology and phenomenology in their study of ambiguous figures. The warning that is presented at the very beginning of their article underlies the arguments developed throughout the paper. This warning concerns the language used in perceptual science, which is sometimes misleading and manifests as conceptual confusions. Such language is sometimes misleading because it suggests that we are measuring what we are observing, while we are not; or because it suggests that we are observing exactly the same as what we are measuring, yet the measurement is carried out in different conditions. In both cases, we confuse something that we know with what we experience. The authors consider apparently dated constructs (Köhler 1929; Bozzi 1972) and demonstrate their topicality using the ambiguous smile of Leonardo da Vinci’s Mona Lisa as a case study. They show that the “sfumato” may well be acknowledged to be the key to the ambiguous smile in both neurophysiological and phenomenological explanations, but while in the former (e.g., Livingstone 2000) it is discussed as generating ambiguity at the level of retinal receptors, in the latter, it is responsible for altering the mode of color appearance and the perceptual belongingness of the slightly darker smudges over the corners of Mona Lisa’s mouth (e.g., Soranzo and Newberry 2015). The authors also show that different assumptions about better emotional perceptions of facial expressions either in peripheral vision or in the center of gaze (again, assumptions based on neurophysiology and phenomenology, respectively), have implications in terms of what is the “true” emotional state attributed to Mona Lisa. For all these reasons, they recommend exploring and integrating the two perspectives.

Zavagno (2023)’s contribution remains within the domain of perceptual science and discusses the conceptualization of illusions. As the author points out, it is fairly easy to agree that the purpose of perception is to gather information about the world, but the story becomes more complicated when we must agree on what we mean by the world. It is at this point that the issue of the veridicality of perception typically comes in. Two mainstream conceptualizations of illusions are presented before introducing a third—phenomenological—stance. The first conceptualization is based on Gibson’s ecological theory (Gibson 1966, 1979), according to which our perceptions mostly correspond to reality. Misperceptions occur sometimes, when the visual information available is qualitatively or quantitatively poor, but these cases do not talk about perception or perceptual processing per se. A second conceptualization of illusions is based on cognitivist approaches to visual perception (e.g., Gregory 1997; Rock 1983), according to which illusions manifest as an incorrect rendering of a distal stimulus due to the visual system’s misleading interpretation of the proximal stimulus, which leads to a wrong representation that does not fit with the physical world. Both perspectives presuppose the idea of veridicality. The third stance, which is suggested by the author, is a phenomenological one (e.g., Zavagno et al. 2015; Savardi et al. 2012). According to this stance, perspective illusions are not considered to be errors. Instead, they manifest a cognitive dissonance between an actual perceptual experience and a hypothesized perception (i.e., something we know about the physical status of the stimuli under observation, that we believe to be what we should actually perceive but do not, and which is ultimately based on an observation carried out in different conditions). According to the author, once the experience of illusion is freed from the veridicality mindset and looked at as an experience of a cognitive clash, rooted in perception, it becomes another interesting opportunity to explore the phenomenal underpinnings of cognitive processes—somewhat in the tradition of intuitive or “naïve” physics (Bozzi 1990a).

Intuitive physics, which is concerned with exploring the psychological foundations of the disparities between intuitive and scientific physics, is the subject of Vicovaro (2023)’s contribution. Research on intuitive physics typically investigates laypeople’s intuitive ideas about a physical object’s speed and trajectory of motion (e.g., when falling, bouncing, swinging, colliding, and the like). As the author points out, there is widespread agreement that intuitive physics is rooted in perceptual experience. What is more controversial is whether perceptual experience is to be considered the factor leading to the inaccurate representation of the physical world manifested in naïve theories and this is the position usually emerging from early studies (e.g., McCloskey et al. 1983; Pittenger 1990), or whether perceptual experience is a truthful source of information and if the systematic errors documented in the early studies are rather related to the abstract and unrealistic tasks used as posited by a recent approach inspired by Bayesian cognitive modeling (e.g., Bass et al. 2021; Fischer and Mahon 2021). A significant body of research both supporting and conflicting with each of the two positions is reviewed in the paper. A special focus is accorded to research indicating that errors occur even when realistic stimuli and scenarios are employed. This leads the author to suggest that a domain-general heuristic (i.e., the idea that people overgeneralize phenomenal—often motor—experiences related to context-specific interactions with objects) can elucidate a wide range of systematic prediction errors that have emerged in the literature.

Metaphors represent another window into the ways in which cognition is grounded in perceptual experience. This topic is addressed by Bracco and Ivaldi (2023) in relation to industrial safety models. Metaphors have been used from the very beginning in organizational risk models to explain how accidents occur (Le Coze 2019). The rationale for doing this is so that a metaphor and its graphical representation can effectively communicate abstract or complex concepts in a more accessible way, by framing organizational failures in concrete terms. However, as the authors make clear by discussing various classic accident models, the use of metaphors and their graphical representations may mislead people’s conceptualization. Although they guide the observers’ minds in framing a domain of knowledge (Refaie 2003), in doing so, they may also foster a selective and incomplete perspective of the accident, accentuating specific features while relegating others to the background, adding wrong assumptions, and begetting temporal or spatial constraints that are linked to representation and not to accident dynamics, thus leading to a biased understanding. As the authors highlight in the concluding section of their paper, the problem is not that accident metaphors reduce the complexity of the reality that they describe—just as maps reduce the complexity of the landscape and are useful exactly for this very reason. Instead, the point is to ensure that this inevitable reduction does not discard essential aspects and does not bias cognition about safety issues, and the challenge is to ensure this by keeping in mind the fact that system complexity is studied by researchers, accidents are investigated by risk managers, and safety is conducted by people working within an organization in their everyday actions. A good model should be able to allow these three parties to share a common perspective. Because of the inter-observability of perceptual experience (Bozzi 1978), graphical representations have this potential on their side.

Inter-observability is also one of the premises in the contribution from Caballero and Paradis (2023). They examine the relationship between perceptual experience and linguistic configuration from the perspective of a contemporary language science approach that is based on embodied cognition (Talmy 2000; Tomasello 2008). They do this by observing how multimodal perceptual experience is communicated through authentic language. The authentic language that is focused on is the language of architects, which is appealing, as architecture is both highly multimodal and highly intermodal (an architecture must be “felt” to be understood). Vision plays a major role too, but it is not the whole story. Moreover, architects are required to talk about their conceptualizations of space (imagined or realized built space) to other architects but also to interlocutors outside the field. The authors analyzed a corpus of texts retrieved from architecture magazines and websites as well as texts produced by architecture students engaged in redesigning a building who were asked to talk aloud while drawing and then provide a final verbal report of their product. The authors derived two main observations from their analysis. First, they noticed many cross-modal expressions, mostly consisting of the following: primarily auditory descriptors that were used to portray visual experiences; visual and tactile experiences that were used to refer to acoustic aspects; and tactile and taste descriptors that were used to refer to sight. Second, with regard to the use of motion language, they revealed that architects frequently portrayed the built space by describing personal experiences while moving around and often talked of space through personification, as if buildings were dynamic entities, with animated properties.

The role of perceptual experience in cognition is examined from yet another viewpoint in the two final articles. Vitello and Salvi (2023) discuss the role that perceptual experience plays in cognition that is focused on problem solving. They concentrate on the phenomenology of the “Aha” experience that often accompanies insight and that involves feelings of surprise, satisfaction, and pleasure (e.g., Danek and Wiley 2017), as well as on the parallelism between the reorganization processes that, according to Gestalt psychologists (Köhler 1925; Wertheimer 1959), occur both in insight problem solving and in figure-ground reversals. The authors review neurophysiological evidence that has emerged in connection with behavioral and self-report measures in recent studies of insight, which seems to support the basic ideas associated with the original Gestalt description of the process. They focus in particular on the findings supporting the link between perceptual and cognitive reorganization, as well as the sudden on-off emergence of the solution to consciousness. Although the revised evidence is not decisive and although it is challenging to capture the shift into awareness that characterizes an insight, the main purpose of the paper is to highlight how researchers have been able to use advancements in techniques to identify physiological measures that might overlap with the behaviorally and phenomenologically described aspects. As the authors put it, if the task is challenging, it is, however, worth posing the question to encourage future investigation.

The final contribution to the Special Issue is by Landmann et al. (2023), who examine the relationship between perceptual experience and cognition within a social cognition framework. They pose the question, if individuals ground their understanding of others’ thoughts and feelings in their own perceptual and factual experiences, does this become a challenge for the possibility to empathize and mentalize with others whose reality of life is significantly different? The article initially looks for an answer in the existing contrasting literature, demonstrating that participants find it easier to take another person’s perspective when they have similar past experiences (e.g., Gerace et al. 2015; Hodges et al. 2010) but, at the same time, that participants can take the perspective of and empathize with outgroup individuals or individuals whose lived situations they themselves could never encounter (e.g., Cao et al. 2015; Van Boven and Loewenstein 2005). The initial question is then addressed by looking for answers in the results of an original study investigating the social understanding of visually impaired and unimpaired participants in relation to stories told by narrators who themselves are either visually impaired or unimpaired. The results of the study suggest that shared visual abilities affect cognitive understanding more than empathy, but they also show that, overall, individuals have the capacity to compensate for discrepancies in perceptual experiences and specific circumstances and derive their social understanding from more basic, fundamental shared experiences and emotions (triggered, for instance, not as much by the content of the narration but by the perceived tone of the voice of the narrator).

Taken together, all the articles in this Special Issue offer a lively picture of the many ways in which a careful analysis of perceptual *experience* stimulates current experimental research. The articles also offer fresh food for thought to enrich, on the one hand, debate related to grounded cognition and embodied theories (Barsalou 2010, 2020; Cowley and Vallée-Tourangeau 2017; Kiefer and Barsalou 2013; Pecher and Zwaan 2005; Varela et al. 2017) and, on the other hand, discussions on the role of phenomenology within the cognitive sciences (Albertazzi 2013; Bianchi and Davies 2019; Gallagher 2012; Gallagher and Zahavi 2008; Ihde 1986; Käufer and Chemero 2015; Kubovy 2002; Mungan 2023).
